# Role of Carbon Monoxide in Oxidative Stress-Induced Senescence in Human Bronchial Epithelium

**DOI:** 10.1155/2022/5199572

**Published:** 2022-09-24

**Authors:** Meng-yun Cai, Chung-Yin Yip, Kewu Pan, Yan Zhang, Renee Wan-Yi Chan, Wood Yee Chan, Wing-Hung Ko

**Affiliations:** ^1^School of Biomedical Sciences, The Chinese University of Hong Kong, N.T., Hong Kong, China; ^2^Department of Paediatrics, The Chinese University of Hong Kong, N.T., Hong Kong, China

## Abstract

Prolonged or excessive stimulation from inhaled toxins may cause oxidative stress and DNA damage that can lead to stress-induced senescence in epithelial cells, which can contribute to several airway diseases. Mounting evidence has shown carbon monoxide (CO) confers cytoprotective effects. We investigated the effects of CO on oxidative stress-induced senescence in human airway epithelium and elucidated the underlying molecular mechanisms. Here, CO pretreatment reduced H_2_O_2_-mediated increases in total reactive oxygen species (ROS) production and mitochondrial superoxide in a human bronchial epithelial cell line (BEAS-2B). H_2_O_2_ treatment triggered a premature senescence-like phenotype with enlarged and flattened cell morphology accompanied by increased SA-*β*-gal activity, cell cycle arrest in G0/G1, reduced cell viability, and increased transcription of senescence-associated secretory phenotype (SASP) genes. Additionally, exposure to H_2_O_2_ increased protein levels of cellular senescence markers (p53 and p21), reduced Sirtuin 3 (SIRT3) and manganese superoxide dismutase (MnSOD) levels, and increased p53 K382 acetylation. These H_2_O_2_-mediated effects were attenuated by pretreatment with a CO-containing solution. SIRT3 silencing induced mitochondrial superoxide production and triggered a senescence-like phenotype, whereas overexpression decreased mitochondrial superoxide production and alleviated the senescence-like phenotype. Air-liquid interface (ALI) culture of primary human bronchial cells, which becomes a fully differentiated pseudostratified mucociliary epithelium, was used as a model. We found that apical and basolateral exposure to H_2_O_2_ induced a vacuolated structure that impaired the integrity of ALI cultures, increased goblet cell numbers, decreased SCGB1A1+ club cell numbers, increased p21 protein levels, and increased SASP gene transcription, consistent with our observations in BEAS-2B cells. These effects were attenuated in the apical presence of a CO-containing solution. In summary, we revealed that CO has a pivotal role in epithelial senescence by regulating ROS production via the SIRT3/MnSOD/p53/p21 pathway. This may have important implications in the prevention and treatment of age-associated respiratory pathologies.

## 1. Introduction

Cellular senescence is defined as an irreversible arrest of cell cycle progression, which occurs either after successive rounds of cellular replication (replicative senescence) or may be triggered by oncogenes and stress (oncogene-induced senescence and stress-induced premature senescence) [1, 2]. The airway epithelium acts as the first line of defense against inhaled toxins (cigarette smoke, harmful microbes, pollutants, and allergens) by recognizing and responding to “danger signals” to repair defects that arise as a result of injury [3]. Meanwhile, prolonged or excessive stimulus from inhaled toxins may cause oxidative stress and induce DNA damage that can lead to stress-induced senescence in epithelial cells. Senescence in airway epithelial cells is notable because these cells are widely recognized for their role in the onset and progression of age-related lung disease, such as chronic obstructive pulmonary disease (COPD) and lung fibrosis [4, 5].

Mitochondria are the main organelle involved in regulating cellular oxidative metabolism. Mitochondrial-derived reactive oxygen species (ROS) are generated in the form of superoxide by complex I and III in the electron transport chain (ETC) as byproducts of inefficient electron transport. In turn, ROS accumulation results in mitochondrial dysfunction and a further decrease in electron transport efficiency, which forms a positive feedback loop of ROS production and oxidative stress that leads to cell dysfunction, including senescence [6]. Sirtuin (SIRT) proteins are a group of epigenetic mediators, which are highly conserved and widely expressed in mammals [7]. There are seven members of SIRT family (SIRT1-7), which participate in various biological processes as nicotinamide adenine dinucleotide- (NAD+-) dependent deacetylases. Among them, SIRT3 is a major mitochondrial deacetylase that plays a pivotal role in response to oxidative stress [8]. The anti-oxidative effects of SIRT3 were mediated via activation of a mitochondrial detoxification enzyme, manganese superoxide dismutase (MnSOD) [9]. *In vivo* and *in vitro* studies have demonstrated SIRT3 is an ROS scavenger and regulator of cellular redox balance. SIRT3 knockout mice were susceptible to aberrant levels of cellular ROS and impaired cellular respiration [10]. Likewise, SIRT3 overexpression mice exhibited decreased ROS levels and mitochondrial oxidative stress [11]. Furthermore, Kong et al. reported SIRT3 is an integrator of energy metabolism and ROS generation at the cellular level [12]. In the lung, SIRT3 inhibits airway epithelial mitochondrial oxidative stress in a cigarette smoke-induced COPD model [13]. Also, SIRT3 mediated fibroblasts-derived extracellular vesicle-induced epithelial cell senescence in pulmonary fibrosis [14]. It was recently reported that SIRT3 could ameliorate lung senescence and improve alveolar epithelial cell function [15].

Inhaled carbon monoxide (CO) is lethal at high concentrations due to CO binding to the heme iron centers of hemoglobin, resulting in asphyxia [16]. Due to its role as a silent asphyxiant, CO was previously described as toxic. Endogenous CO is primarily generated from heme catabolism by two isoforms of heme oxygenase (HO): the constitutively expressed HO-2 and the inducible HO-1 [17]. The traditional notion that CO is solely a toxic chemical has been challenged over the last decade. Not only has this notion been challenged, the protective effects and therapeutic potential of CO have been revealed [18, 19]. Low concentrations of CO are now considered a cyto- and tissue-protector in respiratory and other systems [18, 20]. A series of studies demonstrated that exogenous CO has cytoprotective effects, including anti-oxidation, anti-inflammation, anti-apoptosis, and anti-proliferation [17, 20]. Also, our previous studies revealed the role of CO on cytokine and chloride secretion [21], intracellular calcium regulation [22], and anti-inflammatory activity [23] in bronchial epithelial cells. However, it is unclear whether CO exerts any effect on oxidative stress-induced airway epithelial senescence.

Thus, we investigated whether CO could protect human airway epithelium from oxidative stress-induced senescence and the role of SIRT3.

## 2. Materials and Methods

### 2.1. Cell and Air-Liquid Interface (ALI) Cultures

BEAS-2B cells (a human bronchial epithelial cell line) were generously provided by Prof. Wong. These cells were transformed by adenovirus 12-SV40 virus hybrid (Ad12SV40) and maintain the characteristics of primary human bronchial epithelial cells, which were widely used in the epithelium mechanism studies [24–27]. BEAS-2B cells were cultured in Minimum Essential Medium (MEM) with 5% fetal bovine serum supplemented with 1% L-glutamine and 1% penicillin/streptomycin, in an incubator with humidified atmosphere containing 5% CO_2_ (v/v) at 37°C.

For ALI cultures, HBECs were purchased from ScienCell Research Laboratories (#3210, Carlsbad, CA, USA) and grown in submerged culture in PneumaCult-Ex Plus Expansion Medium (#05040, StemCell, Tukwila WA). Sub-cultured HBECs were seeded on Poly-L-Lysine-coated 24-well Transwell permeable inserts. Expansion medium was applied to the apical and basolateral chambers before the cells reached confluence. After monolayer formation, cells were air-exposed by removing the apical medium, and the basolateral medium was replaced with PneumaCult-ALI Maintenance Medium (#05001, StemCell, Tukwila WA), which was set as the beginning (Day 0; D0) of ALI phase. The basolateral ALI medium was changed every two days, while a pseudostratified layer was obtained, and immunofluorescence staining was performed to observe different cell type markers on ALI-D28.

### 2.2. Preparation of CO-Containing Solution and Detection of Intracellular CO and ROS Levels

Different concentrations of CO-containing solution were prepared as previously described [21]. Briefly, Krebs-Henseleit (KH) solution was saturated with CO gas (99.99%) for 30 min to generate a 100% CO-containing solution. Different concentrations were prepared by diluting the 100% CO-containing stock solution with an unsaturated KH solution. Different cell-permeable fluorescent indicators were used to individually or simultaneously detect intracellular CO and ROS levels in living BEAS-2B cells. A Nile red CO fluorescent probe was generously provided by Prof. Klán and applied to determine the intracellular CO level [28]. BEAS-2B cells were stained with 5 *μ*M CO fluorescent probe and 2 *μ*g/ml Hoechst for 45 min in the presence of different CO concentrations. Intracellular CO level and total ROS production were simultaneously measured by incubating BEAS-2B cells with 20 *μ*M H_2_DCFDA (#C400, Invitrogen, Carlsbad, CA, USA) and fluorescent 5 *μ*M CO probe with 2 *μ*g/ml Hoechst in presence of KH solution or CO-containing solution for 45 min, followed by exposure to 100 *μ*M H_2_O_2_ for 1 h. To determine mitochondrial-derived superoxide (O_2_^•-^) production, CO and H_2_O_2_ treated, or SIRT3 siRNA/overexpression plasmid transfected cells were incubated with 2.5 *μ*M MitoSOX Red (#M36008, Invitrogen, Carlsbad, CA, USA) and 200 nM MitoTracker Green FM (#M7514, Invitrogen, Carlsbad, CA, USA) for 20 min. After staining, the cells were washed twice with PBS and fluorescent images were acquired using an Olympus IX83 Inverted Microscope (Olympus, Tokyo, Japan) within 30 min. The fluorescence levels in each group were normalized by the cell number indicated by Hoechst.

### 2.3. Senescence-Associated *β*-Galactosidase (SA-*β*-Gal) Staining

To observe senescent cells, SA-*β*-gal staining was performed with the Senescence *β*-Galactosidase Staining Kit (#C0602, Beyotime, Shanghai, China) according to the manufacturer's instructions. In brief, PBS washed cells were fixed in fixing buffer for 15 min at room temperature. Then, cells were washed three times with PBS and incubated with working solution at 37°C overnight. Images were acquired using an Olympus IX83 Inverted Microscope (Olympus, Tokyo, Japan). At least six randomly chosen microscopic fields were counted to determine the percentage of SA-*β*-gal-positive cells.

### 2.4. Flow Cytometric Cell Cycle Analysis

Following CO and H_2_O_2_ treatment, BEAS-2B cells were harvested by trypsin digestion then washed with PBS and fixed overnight in 70% ethanol at -20°C. The cells were stained with the Cell Cycle Analysis Kit (#C1052, Beyotime, Shanghai, China) according to the manufacturer's instructions. Briefly, fixed cells were treated with propidium iodide (PI, 50 *μ*g/ml) and RNase A (100 *μ*g/ml) for 30 min at 37°C. 3 x 10^4^ PI labeled cells was evaluated by a FACsort equipped with Cell Quest Software (Becton Dickinson, Palo Alto, CA). Flow cytometry data were analyzed using FlowJo software (FlowJo LLC, Ashland, OR).

### 2.5. Cell Viability Detection (Cell Counting Kit-8, CCK-8 Assay)

BEAS-2B cells were seeded in a 96-well culture plate. After corresponding treatments, 100 *μ*l growth medium containing 10% (v/v) CCK-8 (#C0037, Beyotime, Shanghai, China) was added to each well and incubated for an additional 2 h. The optical density at 450 nm was detected using a microplate reader.

### 2.6. RNA Isolation and RT-qPCR Analysis

RT-qPCR analysis was performed to detect mRNA expression of SASP genes. Total RNA from BEAS-2B cells and ALI cultures was isolated using TRIzol reagent (#15596026, Thermo Fisher Scientific, Waltham, MA, USA) according to the manufacturer's protocol. Total RNA then was reverse transcribed using the PrimeScript RT Reagent Kit with gDNA Eraser (#RR036A, Takara Bio, Shiga, Japan), and RT-qPCR was performed in ABI QuantStudio 7 Pro Real Time PCR System (Applied Biosystems, Carlsbad, CA) with SYBR Green qPCR Master Mix (#A25780, Applied Biosystems, Carlsbad, CA). The specific primers used are listed in Table S1. Relative gene expression was calculated using the 2^–*ΔΔ*CT^ method and *β*-actin was used as internal reference.

### 2.7. Western Blotting

BEAS-2B cells were lysed using RIPA lysis buffer containing protease inhibitors and loading buffer. Total protein was subjected to 12% 8–12% SDS-PAGE electrophoresis and transferred onto a PVDF membrane. The membranes were blocked with 3% fetal bovine serum for 1 h at room temperature, then the membranes were incubated with primary antibodies against p53 (#9282, 1 : 1000, CST), ace-p53 at K382 (#YK0047, 1 : 500, ImmunoWay), p21 (#2947, 1 : 1000, CST), SIRT3 (#2627, 1 : 1000, CST), MnSOD (#sc-137254, 1 : 200, Santa Cruz), or *β*-ACTIN (#sc-8432, 1 : 3000, Santa Cruz) at 4°C overnight. After washing, the membranes were incubated with anti-mouse or anti-rabbit HRP-conjugated secondary antibodies (#ab270144, 1 : 5000, Abcam) for 2 h at room temperature. Protein bands were visualized by chemiluminescence substrate ECL (#1705061, Bio-Red) and captured by the BioRad ChemDoc Imaging System. Protein band intensities were quantified using the ImageJ program and normalized to *β*-ACTIN. Results were expressed as fold increase over control.

### 2.8. Transfection

BEAS-2B were transfected with SIRT3 small interfering RNAs (#sc-61556, siRNAs, Santa Cruz Biotechnologies, South San Francisco, CA) or overexpression plasmid using Lipofectamine RNAiMax and Lipofectamine 2000 (#13778150 and #11668019, Life Technologies, Carlsbad, CA), respectively, according to the manufacturer's protocol.

### 2.9. TEER

TEER of ALI cultures were measured using the Millicell ERS-2 electrical resistance system (Millipore, USA) according to the manufacturer's protocol.

### 2.10. Histological Staining

After 28 days air-lifted with or without the treatment, ALI cultures were fixed in 4% paraformaldehyde for 20 min. Membranes were isolated from the transwell and embedded with 2% agarose gel. After overnight tissue processing, ALI cultures were embedded in paraffin and sectioned. Then, 5 *μ*m sections were stained with H&E or PAS to evaluate general morphology and mucus-producing goblet cells. For immunofluorescence staining, de-paraffinized sections were subjected to antigen retrieval followed by permeabilization with 0.5% Triton X-100 in PBS and blocking with 3% BSA +0.1% Triton X100 in PBS. Primary antibodies, p21 (#2947, CST, 1 : 200), *α*-tubulin (#T7451, sigma, 1 : 1000), and SCGB1A1 (#A1730, ABclonal, 1 : 100), were diluted in blocking buffer and incubated with the ALI sections at 4°C in a humidified chamber overnight. Primary antibody incubated sections were washed and secondary antibody (#A32723, Alexa Flour-488 conjugated and #A32733, Alexa Flour-647 conjugated, Invitrogen, 1 : 1000) and MUC5AC (#ab218363, Alexa Fluor-594 conjugated, abcam, 1 : 100) were incubated at room temperature for 1 h in a dark humidified chamber. After wash, sections were counterstained with Hoechst. Images were acquired using an Olympus IX83 Inverted Microscope (Olympus, Tokyo, Japan).

### 2.11. Data Analysis

All data were expressed as mean ± SEM. Comparison of the means was performed using Student's *t*-test, one-way ANOVA, and two-way ANOVA followed by Tukey's multiple comparisons test, using the Graph Pad Prism 8 Software (Prism, San Diego, CA). Any difference with *p* values ≤ 0.05 was considered significant.

## 3. Results

### 3.1. CO Suppressed H_2_O_2_-Induced Increased Intracellular ROS and Mitochondrial Superoxide in Airway Epithelial Cells

**Figure 1:**
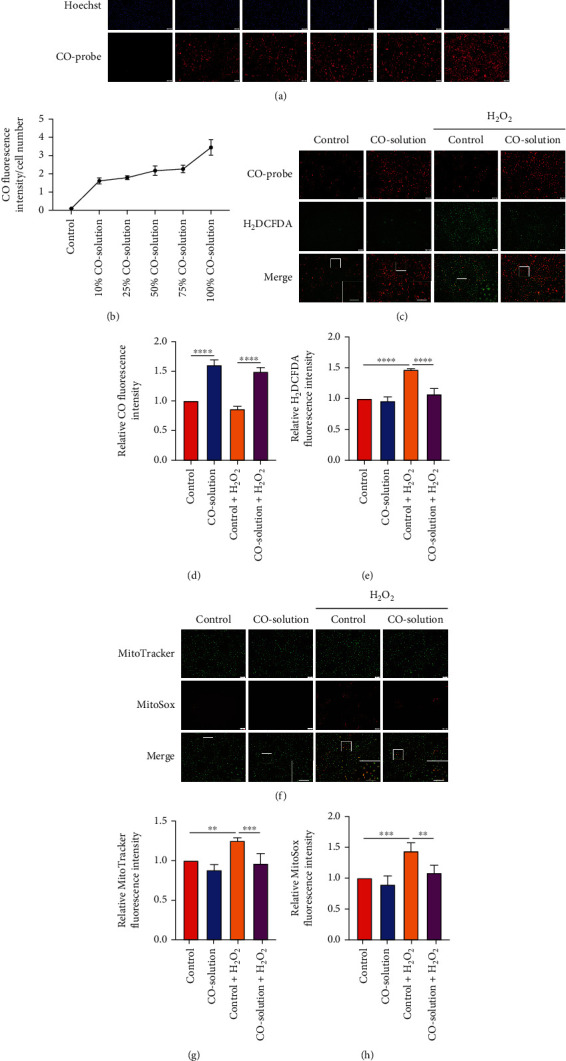
CO suppressed H_2_O_2_-induced increase in intracellular total ROS and mitochondrial superoxide in airway epithelial cells. (a, c, and f) Representative fluorescent images and (b, d, e, g, and h) quantification of intracellular CO and ROS levels in live BEAS-2B cells. (a and b) Cells were stained with CO probe (red) and Hoechst (blue) for 45 min in the presence of different concentrations (0-100%) of CO-containing solution. BEAS-2B cells were treated with (c–e) carboxy-H_2_DCFDA (green), CO probe (Red), or (f–h) MitoTracker (green) MitoSOX (red) in control (KH solution) or 50% CO-containing solution, followed by exposing to 100 *μ*M H_2_O_2_ for 1 h. The fluorescence level in each group was normalized by the cell number indicated by Hoechst. The data were analyzed by one-way ANOVA followed by Tukey's multiple comparisons test, and expressed as the mean ± SEM (*n* =4). ^∗∗^*p* < 0.01; ^∗∗∗^*p* < 0.001; ^∗∗∗∗^*p* < 0.0001.

Because the biological effects of CO are highly dependent on its intracellular concentration, there are several methods to detect intracellular CO level, including infrared, electrochemical, colorimetric, and fluorescent molecular chemosensors [29–32]. In the current study, we applied a fluorescent molecular chemosensor, Nile red CO fluorescent probe [28] to determine the intracellular CO level. There was a dose-dependent increase in the fluorescence level in the presence of different concentrations (0, 10, 25, 50, 75, 100%) of CO-containing solution compared to the control solution (Figures 1(a) and 1(b)), suggesting this CO probe can semi-quantify the intracellular CO level in living cells. To determine the optimal concentration of hydrogen peroxide (H_2_O_2_) and CO for airway epithelial cell treatment, we performed a CCK-8 assay to detect cell viability using different concentrations of H_2_O_2_ or CO. As shown in Figure S1a, a relative decrease in cell viability emerged at 100 *μ*M H_2_O_2_, though more cell death was observed at the concentrations of 150 and 200 *μ*M. Furthermore, there was no significant difference in relative cell viability with different concentrations of CO-containing solution (range from 25% to 100%), as shown in Figure S1b. Of all the tested CO-containing solution concentrations, cells pretreated with 50% CO-containing solution showed greater viability after 100 *μ*M H_2_O_2_ treatment. Thus, 100 *μ*M H_2_O_2_ and 50% CO-containing solution were determined as optimal concentrations for the subsequent study. We previously reported the concentration of CO in the 50% CO-containing solution was 207.31 ± 20.17 *μ*M as determined by a myoglobin assay [21]. Carboxy-H_2_DCFDA (H_2_DCFDA) can be used to detect total ROS levels. H_2_DCFDA is non-fluorescent until intracellular oxidation by ROS removes the acetate groups, which results in conversion to DCF, which is fluorescent [33]. CO probe and H_2_DCFDA co-staining allow simultaneous measurement of CO and ROS levels in living cells. Compared to the control group, the presence of the 50% CO-containing solution resulted in an increase in fluorescence intensity (Figures 1(c) and 1(d)). Concurrently, the H_2_O_2_-mediated increase in total intracellular ROS production was reduced by CO treatment (Figures 1(c) and 1(e)). Given mitochondria are an important source of ROS production, we examined whether CO treatment could inhibit H_2_O_2_-induced mitochondrial ROS production. We used two other cell-permeable fluorescent indicators, MitoSOX [34] and MitoTracker [35], to measure mitochondrial-derived superoxide (O_2_^•-^) and to localize mitochondria, respectively. As shown in Figures 1(f) and 1(g), MitoTracker fluorescence intensity increased after H_2_O_2_ exposure but decreased with CO pretreatment. This increase in mitochondria was consistent with previous findings and may be a response oxidative stress [36]. Likewise, the H_2_O_2_-mediated increase in mitochondrial-derived O_2_^•-^ was suppressed by the 50% CO-containing solution treatment (Figures 1(f) and 1(h)).

### 3.2. CO Attenuated H_2_O_2_-Induced Cellular Senescence

**Figure 2:**
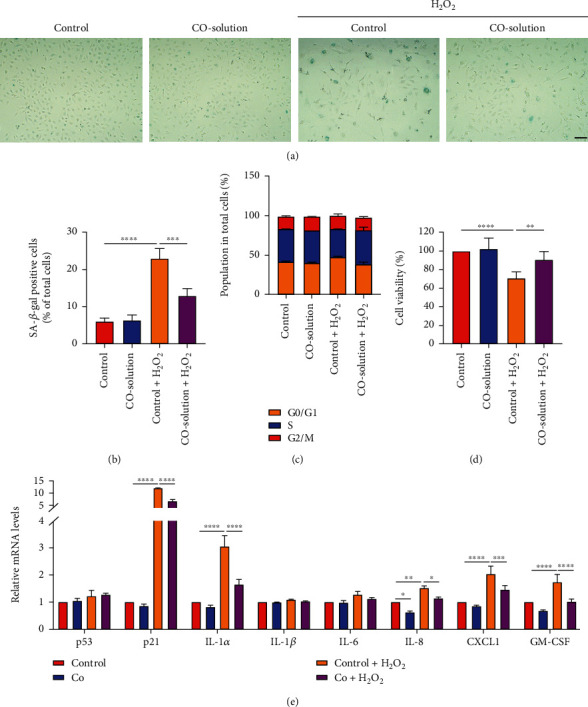
CO attenuated H_2_O_2_-induced cellular senescence. (a) Representative images and (b) quantification data of SA-*β*-galactosidase positive (blue) BEAS-2B cells pretreated with control or 50% CO-containing solution followed by H_2_O_2_ exposure for 96 h. (c) Flow cytometric cell cycle distribution assay to detect the proportion of BEAS-2B cells in G1, S, and G2/M phases and (d) CCK-8 assay to detect cell viability for cells pretreated with a control or 50% CO-containing solution followed by H_2_O_2_ exposure for 48 h. (e) mRNA expression of SASP genes was detected by RT-qPCR with pretreated with control, 50% CO-containing solution followed by H_2_O_2_ exposure for 12 h. The data were analyzed by one-way ANOVA (b and d) or two-way ANOVA (c and e) followed by Tukey's multiple comparisons test and expressed as the mean ± SEM (*n* =3). ^∗^*p* < 0.05; ^∗∗^*p* < 0.01; ^∗∗∗^*p* < 0.001; ^∗∗∗∗^*p* < 0.0001.

Given the established role of oxidative stress in regulating the onset and maintenance of cellular senescence, we hypothesized CO may protect against H_2_O_2_-induced epithelial cell senescence. Compared to the control group, exposure of BEAS-2B cells to H_2_O_2_ triggered an enlarged but flattened cell morphology accompanied with an increased number of SA-*β*-Gal-positive cells (Figures 2(a) and 2(b)). Furthermore, cell cycle analysis revealed H_2_O_2_ treatment increased the number of cells in G1 phase (*p* < 0.05) but decreased the number of cells in S phase (*p* < 0.05) (Figure 2(c)). In addition, H_2_O_2_ exposure reduced cell viability (Figure 2(d)). Critically, 1 h pretreatment with 50% CO-containing solution reversed all of these effects. The senescence-associated secretory phenotype (SASP) includes expression of several cytokines, chemokines, and growth factors secreted by senescent cells. To determine the suppressive effects of CO on SASP, we analyzed the mRNA levels of eight senescence-related genes. As shown in Figure 2(e), CO-containing solution pretreated cells exhibited reduced expression of p21, IL-8, CXCL1, and GM-CSF in response to H_2_O_2_.

### 3.3. The Anti-Senescent Effects of CO Are Mediated by SIRT3

**Figure 3:**
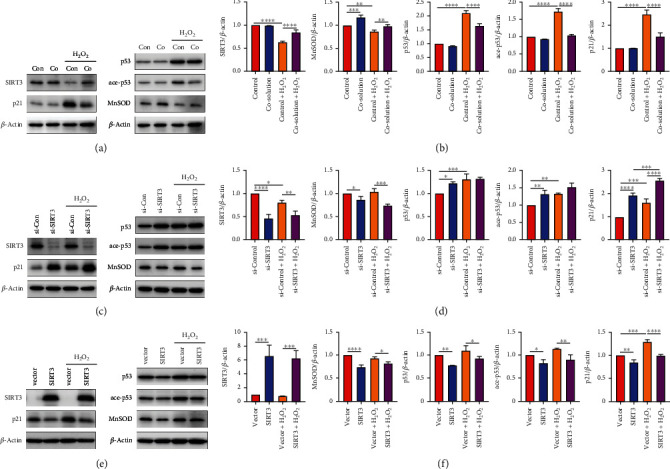
SIRT3 mediated the inhibitory effects of CO on H_2_O_2_-induced expression of senescence markers. (a, c, and e) Representative western blots and (b, d, and f) quantification data showing protein expression levels. (a and b) BEAS-2B cells pretreated with control or 50% CO-containing solution followed by H_2_O_2_ exposure for 48 h. (c–f) BEAS-2B cells were transfected with SIRT3 siRNA or overexpression plasmid for 48 h followed by H_2_O_2_ exposure for 24 h. *β*-ACTIN was used as loading control for all experiments. The data were analyzed by one-way ANOVA followed by Tukey's multiple comparisons test, and expressed as the mean ± SEM (*n* =3). ^∗^*p* < 0.05; ^∗∗^*p* < 0.01; ^∗∗∗^*p* < 0.001; ^∗∗∗∗^*p* < 0.0001.

Previous studies demonstrated the anti-oxidative and anti-senescence effects of SIRT3 in airway epithelial cells. Previous reports demonstrated SIRT3 mediates the protective effect of hydrogen by inhibiting ROS-induced increased expression of p53 and p21, which are cell cycle regulatory proteins and potent markers of cellular senescence [37]. Therefore, we sought to investigate whether SIRT3 mediates the effects of CO. As shown in Figures 3(a) and 3(b), exposure to 50% CO-containing solution prior to H_2_O_2_ treatment in BEAS-2B cells prevented increased expression of p53 and p21, which was consistent with the senescent phenotypes demonstrated in Figure 2. Interestingly, CO pretreatment also reversed the decreased expression of SIRT3 and MnSOD in cells treated with H_2_O_2_. It was recently reported that SIRT3 deacetylates p53, which reduces p53 occupancy of mitochondrial DNA thereby decreasing oxidative stress [38]. We also found CO could reduce p53 acetylation (ace-p53) at K382.

Next, we performed gain and loss of function studies to further elucidate the role of SIRT3 in H_2_O_2_-induced oxidative stress in epithelial cells. SIRT3 silencing by small interfering RNA (siRNA) transfection reduced MnSOD expression and increased p21, p53, and ace-p53 expression (Figures 3(c) and 3(d)), which were augmented by H_2_O_2_ treatment. Conversely, p21, p53, and ace-p53 (Figures 3(e) and 3(f)) expression levels were decreased in BEAS-2B cells transfected with a SIRT3 overexpression plasmid. Unexpectedly, MnSOD expression decreased following SIRT3 overexpression (Figures 3(e) and 3(f)).

### 3.4. SIRT3 Mediated the Anti-Oxidative Effects of CO

**Figure 4:**
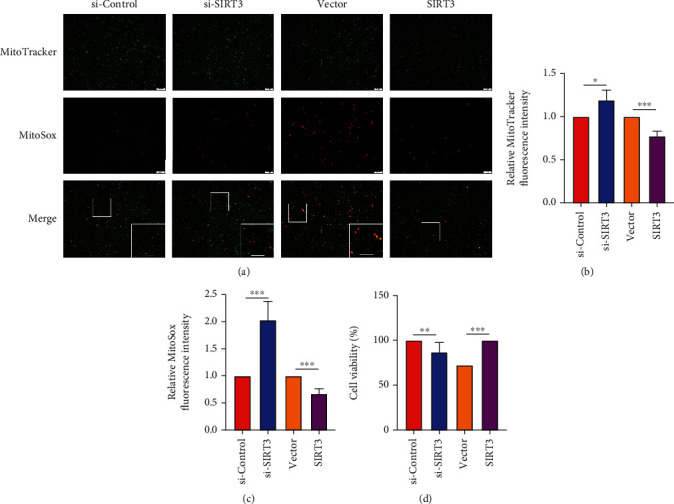
SIRT3 regulated mitochondrial superoxide production and airway epithelial cell viability. BEAS-2B cells were transfected with a SIRT3 siRNA or overexpression plasmid for 48 h (a–c), then stained with MitoTracker (green) and MitoSOX (red) to measure mitochondrial superoxide production and (d) CCK-8 assay to measure cell viability. The data were analyzed by Student's *t*-test and expressed as the mean ± SEM (*n* =4). ^∗∗^*p* < 0.01; ^∗∗∗^*p* < 0.001.

To further elucidate the regulatory effects of SIRT3 on mitochondrial-derived O_2_^•-^ production and cell viability, BEAS-2B cells with SIRT3 knocked down and overexpressed were co-stained with MitoTracker and MitoSox or incubated with CCK-8 reagent. As expected, SIRT3 knockdown triggered an increase in mitochondrial-derived O_2_^•-^ (Figures 4(a)–4(c)) and reduced cell viability (Figure 4(d)). Conversely, mitochondrial-derived O_2_^•-^ (Figures 4(a)–4(c)) was decreased, which was accompanied by increased cell viability (Figure 4(d)) in SIRT3 overexpressing BEAS-2B cells.

### 3.5. CO Attenuated H_2_O_2_-Induced Structural Impairment and Senescent Phenotypes in ALI Cultures

**Figure 5:**
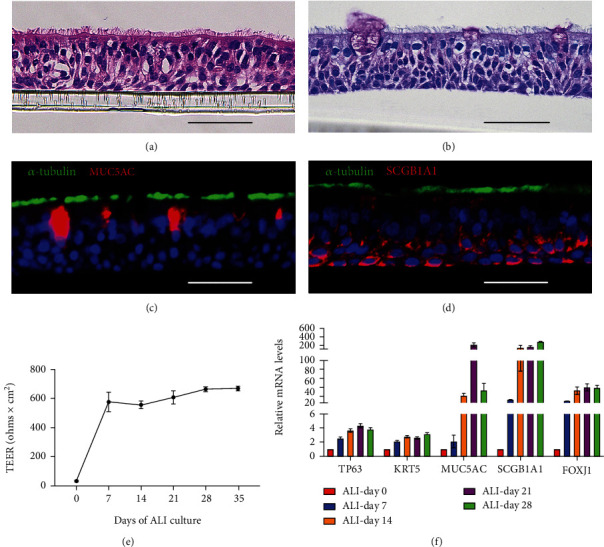
Characterization of mucociliary phenotype and cell-specific markers of ALI cultures derived from primary HBECs. (a) Hematoxylin & Eosin (H&E) staining and (b) Periodic acid Schiff (PAS) staining demonstrated mucociliary phenotype of the ALI cultures at day 28. These multilayered pseudostratified epithelium models were composed of at least ciliated cells and goblet cells located on the apical side. Immunofluorescence for cell-specific markers of (c) ciliated cells (*α*-tubulin, green)/goblet cells (MUC5B, red), (d) ciliated cells (*α*-tubulin, green)/club cells (SCGB1A1, red), and nuclear counterstain (Hoechst, blue). (e) Transepithelial electrical resistance (TEER) and (f) mRNA levels of epithelial cell type-specific markers (TP63, KRT5, MUC5AC, SCGB1A1, FOXJ1) were detected each week after being air-lifted. The data were expressed as the mean ± SEM. Scale bar is equal to 50 *μ*m.

At day 28, we used Hematoxylin & Eosin (H&E) staining and Periodic acid Schiff (PAS) to evaluate the pseudostratified mucociliary structure (Figures 5(a) and 5(b)). Cell type-specific markers were detected by immunofluorescence staining. Ciliated cells (*α*-tubulin)/goblet cells (MUC5B) and ciliated cells (*α*-tubulin)/club cells (SCGB1A1) were identified in the 28 days ALI cultures (Figures 5(c) and 5(d)). Furthermore, as shown in Figures 5(e) and 5(f), transepithelial electrical resistance (TEER) and epithelial cell type-specific markers TP63 (basal cell), KRT5 (basal cell), MUC5AC (goblet cell), SCGB1A1 (club cell), and FOXJ1 (ciliated cell) increased during 28 days of ALI culture, suggesting the ALI cultures were well-differentiated and a suitable model to study the effects of CO on airway epithelial cells.

**Figure 6:**
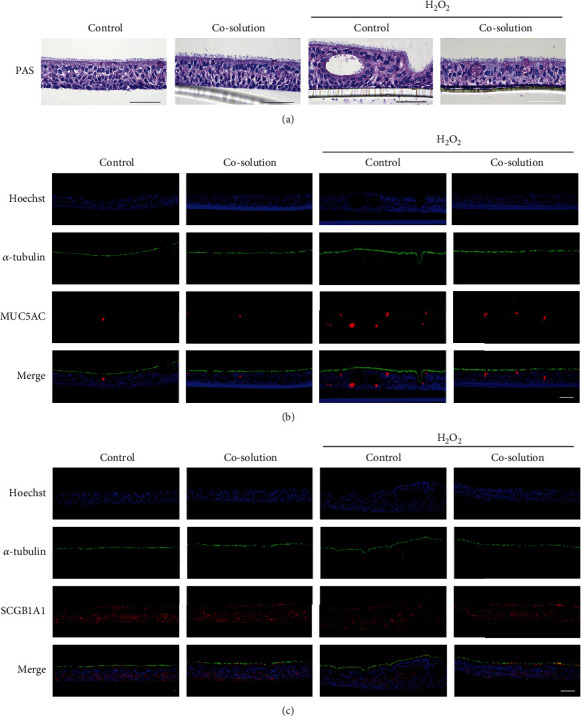
CO attenuated H_2_O_2_-induced structural impairment and change of cell type-specific markers in ALI cultures. (a) PAS staining showed mucus-producing goblet cells and a vacuolated structure upon H_2_O_2_ treatment. Immunofluorescence for cell-specific markers of (b) ciliated cells (*α*-tubulin, green)/goblet cells (MUC5B, red) and (c) ciliated cells (*α*-tubulin, green)/club cells (SCGB1A1, red) with nuclear counterstain (Hoechst, blue). Scale bar is equal to 100 *μ*m.

Differentiated ALI cultures were apically pretreated with a control or 50% CO-containing solution for 30 min, followed by apical and basolateral exposure to 200 *μ*M H_2_O_2_ for 12 h. The treatment was repeated every other day for a total of four times. The samples were collected at day 8 after the first treatment. Results from PAS staining showed that H_2_O_2_ exposure induced a vacuolated structure and impaired ALI culture integrity. However, the ALI cultures treated with CO prior to H_2_O_2_ just showed increase in mucus secreting goblet cell and a relative intact structure was maintained (Figure 6(a)). To detect the effects of H_2_O_2_ and CO on different cell types, cell-specific marker was detected after the treatment. In consistent with the results from the PAS staining, a vacuolated structure was indicated by the discrete fluorescence signal of Hoechst in the ALI cultures exposed to H_2_O_2_ (Figures 6(b) and 6(c)). Additionally, cell type-specific marker of goblet cells, MUC5AC was increased after H_2_O_2_ treatment (Figure 6(b)). However, the number of SCGB1A1+ club cells, which are responsible for repairing damage in airway epithelium, was decreased after H_2_O_2_ treatment (Figure 6(c)). Similarly, these effects were partially reversed in the presence of the CO-containing solution.

**Figure 7:**
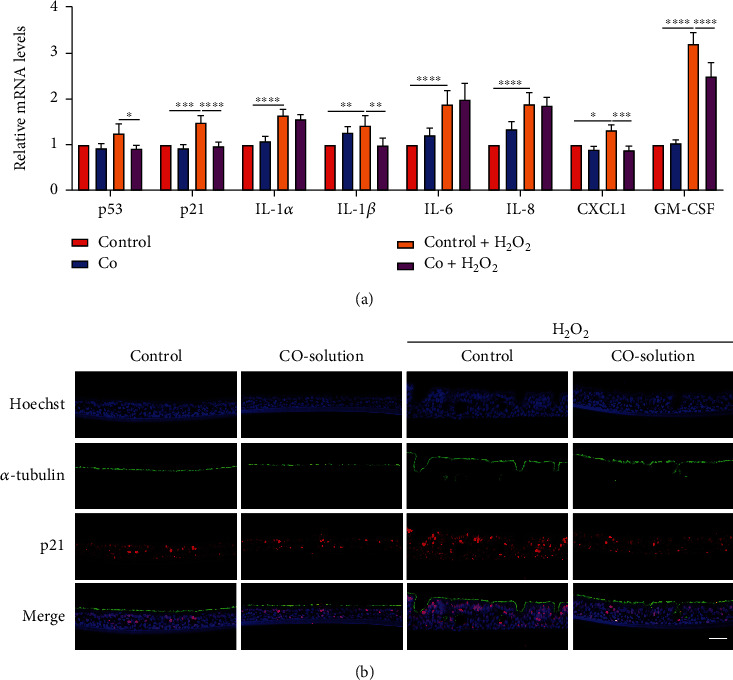
CO attenuated H_2_O_2_-induced increase in SASP and p21 in ALI cultures. (a) mRNA expression of SASP genes was detected by RT-qPCR from ALI cultures exposure to 50% CO-containing solution and H_2_O_2_ for 12 h. The data were analyzed by two-way ANOVA followed by Tukey's multiple comparisons test, and expressed as the mean ± SEM (*n* =4). ^∗^*p* < 0.05; ^∗∗^*p* < 0.01; ^∗∗∗^*p* < 0.001; ^∗∗∗∗^*p* < 0.0001. (b) Immunofluorescence for ciliated cells (*α*-tubulin, green)/senescence marker (p21, red), with nuclear counterstain (Hoechst, blue) after exposure to in total 4 times of 50% CO-containing solution and H_2_O_2_. Scale bar is equal to 100 *μ*m.

To evaluate the effect of CO on H_2_O_2_-induced senescence, measurement of SASP and p21 immunofluorescence staining were performed. Immunofluorescence showed that H_2_O_2_ treatment increased SASP gene transcription (Figure 7(a)) and p21 levels at both apical and basolateral sides (Figure 7(b)). These effects were also attenuated by CO pretreatment, which was consistent with the observations in BEAS-2B cells. These data suggested that CO might also attenuate oxidative stress-induced senescence in ALI-cultured human airway epithelium.

## 4. Discussion

Accumulation of senescent cells is presumed to contribute to age-related diseases pathogenesis by at least two distinct mechanisms. First, senescent cells are increasingly considered malignant “zombie cells” because they are resistant to cell death but are unable to proliferate to repair tissue defects. Second, senescent cells act as pro-inflammatory mediators with the capacity of inducing chronic inflammation and subsequent tissue dysfunction [39–41]. An accumulation of senescent airway epithelial cells has been found in the lungs of chronic lung disease patients, including COPD and idiopathic pulmonary fibrosis (IPF) [42, 43]. Therefore, an understanding of the molecular mechanism can be used to intervene in the process of airway epithelial senescence, which may provide new strategies for the prevention and treatment of age-related respiratory diseases. To our knowledge, the current study is the first to provide evidence suggesting exogenous application of CO protects the airway epithelium from oxidative stress-induced intracellular ROS production and senescence in bronchial epithelial cells and ALI-cultured epithelium models. The data presented here demonstrated CO exerts a protective effect by inhibiting H_2_O_2_-induced intracellular ROS formation via the SIRT3/p53/p21 pathway.

In the cell, senescence can be triggered when cells are exposed to oxidative stress, and H_2_O_2_ is the most commonly used inducer of stress-induced premature senescence (SIPS) [44]. As a result of H_2_O_2_ exposure, DNA damage and double-strand breaks can occur, resulting in overactivity of ADP-ribose polymerase, as well as depletion of NAD+ and ATP. Further, excessive ROS production by H_2_O_2_ exposure inhibits antioxidant activity, further increasing the production of endogenous free radicals. As a result of these changes, SIPS develops morphological, biomolecular, and genetic alterations [45]. H_2_O_2_ exposure affects premature senescence differently depending on the cell type, exposure time, dose, and solvent used [46, 47]. We tested different concentrations of H_2_O_2_ to determine the most effective concentration to establish a premature senescence model since there is no fixed protocol for H_2_O_2_ exposure. When H_2_O_2_-induced premature senescence of fibroblasts was studied, the proportion of SA-*β*-Gal-positive cells increased significantly after treatment with 25 and 50 *μ*M H_2_O_2_. The percentage decreased, however, in fibroblasts treated with 100 *μ*M or higher H_2_O_2_, presumably due to a sharp reduction in cell numbers [46]. We observed the same results in BEAS-2B cells. In general, H_2_O_2_-induced premature senescence is a chronic process that involves low concentrations and continuous processes, whereas high concentrations and transient stimulation cause apoptosis or necrosis instead of senescence.

Though ROS production can lead to senescence, ROS production is also a consequence of senescence. Excessive oxidative stress can cause DNA damage, leading to cellular senescence. On the other hand, the accumulation of dysfunctional mitochondria and other organelles within senescent cells might in turn promote oxidative stress and enhance intracellular ROS production [41, 48]. In the present study, we simultaneously measured intracellular CO and ROS levels and verified that CO negatively regulated H_2_O_2_-induced ROS production, which might contribute to the resulting airway epithelial cell senescence. The mitochondria and the nicotinamide adenine dinucleotide phosphate (NADPH) oxidase (NOX) family are the major sources of intracellular ROS [49]. Evidence suggests that the anti-oxidative properties of CO may be due to its ability to bind to heme-containing proteins, NOX family members, and mitochondrial complexes of ETC, which are responsible for ROS production after oxidative stress [50, 51]. As reported previously, CORM-2 inhibits NADPH oxidase cytochrome b558 and respiratory chain activity in complex I of airway smooth muscle cells to inhibit proliferation [50]. Nitric oxide is produced by endothelial nitric oxide synthase in vascular endothelium, which directly or indirectly regulates mitochondrial ROS production [52]. Additionally, the metal-binding nature of CO suggests that cyclooxygenase, cytochrome p450, and cytochrome c oxidase could also serve as CO sensors [51]. Therefore, we also demonstrated that CO inhibited H_2_O_2_-induced mitochondrial-derived superoxide production, which is consistent with results from other studies using different cell types [33, 53].

SIRT3 plays a critical role in mitochondrial metabolism and is mainly localized in the mitochondrial matrix [54]. Mitochondrial dysfunction is closely associated with several respects of senescence, including reduced metabolic enzyme activity and increased oxidative damage [55]. SIRT3 is a demonstrated major risk factor of senescence and aging as decreased SIRT3 expression is frequently observed in many age-related diseases [56]. It has been reported that SIRT3 deficiency promoted pulmonary fibrosis [57, 58]. More recent studies have demonstrated SIRT3 can prevent airway epithelial mitochondrial oxidative stress in cigarette smoke-induced COPD, possibly through the regulation of MnSOD expression levels and activity [13]. In the current study, we first demonstrate that SIRT3/MnSOD mediated the anti-senescent effects of CO in H_2_O_2_-induced oxidative stress in airway epithelium cells. A previous study discovered SIRT3 regulated mitochondrial gene expression, mitochondrial oxygen consumption, and ROS production in Alzheimer's disease by reducing p53 acetylation levels. Consistent with this report, we found SIRT3 attenuated H_2_O_2_-induced increased ROS production and p53 acetylation levels, as well as expression of its downstream protein, p21. Though SIRT3 overexpression unexpectedly decreased MnSOD levels, it still exerted anti-senescent and anti-oxidative effects. This result is likely due to the positive feedback inhibition and effects of MnSOD, which depends on its acetylation level and activity in addition to its expression level [59, 60].

The ALI culture technique provides a mucociliary model to study lung biology that recapitulates normal airway epithelium biology *in vitro* [61]. Optimal and stable differentiation of ALI cultures was achieved from commercial human primary bronchial epithelial cells (HBECs) and culture medium in the current study. The expression of cell type-specific markers and senescence-associated genes was evaluated after treatments. MUC5AC upregulation is an important characteristic of goblet cell metaplasia and mucus hypersecretion, which is always observed in airway inflammation diseases, such as asthma and COPD [62–65]. SCGB1A1, also known as CC10, is a member of the secret globin family, which is mainly produced by club cells [66]. SCGB1A1 is especially abundant in the peripheral airways and patients with asthma, COPD, and obliterative bronchiolitis after lung transplantation have deficient SCGB1A1 expression in their airways [67–70]. These observations suggest club cells and SCGB1A1 play an important role in the control of the airway integrity and repair response upon injury. Indeed, several studies have demonstrated SCGB1A1 may be responsible for exerting club cell effects, such as immunomodulation, inhibiting mucus secretion, and modulating epithelial remodeling. Moreover, there are *in vivo* studies demonstrating that SCGB1A1+ club cells possessed self-renewal capacity and could give rise to new ciliated cells after lung injury, where they contribute to tracheal repair [71, 72]. The results of our current study showed H_2_O_2_ treatment increased the number of MUC5AC+ goblet cells but decreased the number of SCGB1A1+ club cells. Ineffective club cell restoration, which is likely due to oxidative stress-induced senescence in club cells, reduced SCGB1A1 expression. In turn, this contributed to mucus secretion, increased p21 protein levels, and increased SASP gene transcription. These effects were attenuated in the presence of the CO-containing solution.

## 5. Conclusions

**Figure 8:**
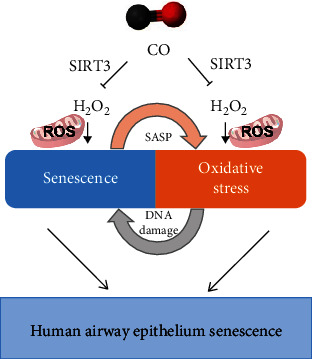
Schematic summary of the role and underlying mechanisms of CO in oxidative stress-induced senescence in human airway epithelium. The oxidative stress might lead to cellular senescence by causing DNA damage. In turn, the senescent cells could also augment oxidative stress by SASP and form a vicious cycle. The present study verified that H_2_O_2_-induced airway epithelium senescence, and the effects could be partially reversed by CO. This protective mechanism, at least in part, by inhibiting the mitochondrial ROS production and mediated by the mitochondrial deacetylase SIRT3.

Overall, the present study verified that CO protects the airway epithelium from H_2_O_2_-induced senescence. From the data presented here, CO exerts a protective mechanism at the cellular levels by inhibiting intracellular ROS formation by activating the SIRT3/MnSOD/p53/p21 pathway (Figure 8). However, CO might play different roles in ALI cultures depending on cell type. These findings improve our understanding of the underlying molecular mechanisms of CO in oxidative stress-induced senescence, which may contribute to age-related lung disease pathogenesis. However, further *in vivo* validation and detailed mechanistic studies are required to establish the clinical potential of CO in age-related airway diseases.

## Data Availability

The datasets used to support the current study are available from the corresponding author upon reasonable request.

## Supplementary Materials

Supplementary Materials Table S1: Oligomers used in this study. Figure S1: CCK-8 assay used to detect cell viability with treatment of different concentrations of (a) H_2_O_2_ or (b) CO with or without H_2_O_2_ exposure for 24 h.
