# Supplementary material for: Role of Carbon Monoxide in Oxidative Stress-Induced Senescence in Human Bronchial Epithelium
Source: Oxid Med Cell Longev. 2022 Sep 24;2022:5199572. doi: 10.1155/2022/5199572 (PMC9526622; doi:10.1155/2022/5199572)
Supplement: Supplementary Materials — Table S1: Oligomers used in this study. Figure S1: CCK-8 assay used to detect cell viability with treatment of different concentrations of (a) H2O2 or (b) CO with or without H2O2 exposure for 24 h. [file 5199572.f1.zip › Supplementary Table S1.docx]

**Supplementary Table S1 Oligomers used in this study**

| **Oligomer Name** | **Sequence (5’-3’)**  **Forward** | **Sequence (5’-3’)**  **Reverse** |
| --- | --- | --- |
| β-actin | AAGATGACCCAGATCATGTTTGAG | GCAGCTCGTAGCTCTTCTCCAG |
| p21 | TGTCCGTCAGAACCCATGC | AAAGTCGAAGTTCCATCGCTC |
| p53 | CAGCACATGACGGAGGTTGT | TCATCCAAATACTCCACACGC |
| IL-1α | TATGGCTCCACCCAGAAG | AGCTCATAGGTTAGGGATTTT |
| IL-1β | CTTCCAGTGGATTTCCTTGC | CATCTTGAGGGGCATCTTTT |
| IL-6 | AATTCGGTACATCCTCGACGG | GGTTGTTTTCTGCCAGTGCC |
| IL-8 | AACTTCTCCACAACCCTCTG | TTGGCAGCCTTCCTGATTTC |
| GM-CSF | CTTCCTGTGCAACCCAGATT | CAGCAGTCAAAGGGGATGAC |
| KRT5 | CTCTGGATATGGCAGTGGCAG | CTGCTGCTGGAGTAGTAGCTT |
| TP63 | CCCGTTTCGTCAGAACACAC | CATAAGTCTCACGGCCCCTC |
| MUC5AC | GGAGGTGCCCTTCAGCAA | CGTGCGGCACTCATCCTT |
| SCGB1A1 | ATGAAACTCGCTGTCACCCT | GGGTTTCGATGACACGCTGA |
| FOXJ1 | CTTTCAAGAAGCGGCGACTG | CCTCGGTATTCACCGTCAGC |
