# Supplementary figures and images for: Role of Carbon Monoxide in Oxidative Stress-Induced Senescence in Human Bronchial Epithelium
Source: Oxid Med Cell Longev. 2022 Sep 24;2022:5199572. doi: 10.1155/2022/5199572 (PMC9526622; doi:10.1155/2022/5199572)

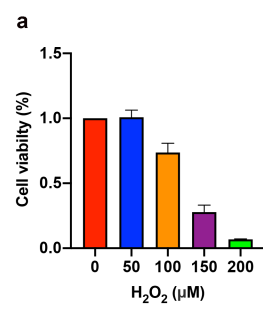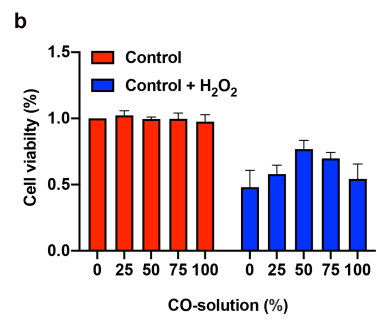

Supplement: Supplementary Materials — Table S1: Oligomers used in this study. Figure S1: CCK-8 assay used to detect cell viability with treatment of different concentrations of (a) H2O2 or (b) CO with or without H2O2 exposure for 24 h. [file 5199572.f1.zip › Figure S1.pdf]
